# The σ^54^ system directly regulates bacterial natural product genes

**DOI:** 10.1038/s41598-021-84057-4

**Published:** 2021-02-26

**Authors:** Muqing Ma, Roy D. Welch, Anthony G. Garza

**Affiliations:** Department of Biology, Syracuse University, 107 College Place, Syracuse, NY 13244 USA

**Keywords:** Biotechnology, Microbiology, Molecular biology

## Abstract

Bacterial-derived polyketide and non-ribosomal peptide natural products are crucial sources of therapeutics and yet little is known about the conditions that favor activation of natural product genes or the regulatory machinery controlling their transcription. Recent findings suggest that the σ^54^ system, which includes σ^54^-loaded RNA polymerase and transcriptional activators called enhancer binding proteins (EBPs), might be a common regulator of natural product genes. Here, we explored this idea by analyzing a selected group of putative σ^54^ promoters identified in *Myxococcus xanthus* natural product gene clusters. We show that mutations in putative σ^54^-RNA polymerase binding regions and in putative Nla28 EBP binding sites dramatically reduce in vivo promoter activities in growing and developing cells. We also show in vivo promoter activities are reduced in a *nla28* mutant, that Nla28 binds to wild-type fragments of these promoters in vitro, and that in vitro binding is lost when the Nla28 binding sites are mutated. Together, our results indicate that *M*. *xanthus* uses σ^54^ promoters for transcription of at least some of its natural product genes. Interestingly, the vast majority of experimentally confirmed and putative σ^54^ promoters in *M*. *xanthus* natural product loci are located within genes and not in intergenic sequences.

## Introduction

The σ^54^ regulatory system modulates transcription of a wide variety of bacterial genes. One crucial component of this regulatory system is the σ^54^ protein, which directs RNA polymerase to conserved DNA sequences located in the − 12 and − 24-bp regions of σ^54^ promoter elements^1,2^. Enhancer binding proteins (EBPs), which are transcriptional activators, are also crucial for the normal function of the σ^54^ regulatory system. Namely, EBPs are ATPases that use the energy from ATP hydrolysis to help σ^54^-RNA polymerase form an open promoter complex and initiate transcription^3–5^. Bacteria typically have one gene for σ^54^, but often have multiple genes for EBPs; each EBP works with σ^54^ to regulate a subset of σ^54^ promoters, which the EBP identifies via specific tandem repeat sequences or enhancer elements^6,7^. Interestingly, the tandem repeat binding sites of EBP dimers are typically located 80- to 150-bp upstream of the − 12 and − 24-bp regions of σ^54^ promoters; hence, it seems likely that many σ^54^ promoters have intrinsically curved DNA sequences or binding sequences for DNA bending proteins, as EBP dimers directly contact σ^54^-RNA polymerase^8–10^.

EBPs generally contain three domains: an N-terminal signaling domain, a central ATPase domain that is responsible for ATP hydrolysis and transcriptional activation, and a C-terminal DNA-binding domain (DBD) that recognizes a specific DNA sequence^7^. Typically, the N-terminal signaling domain modulates the ATPase activity of the EBP in response to an intracellular or extracellular signal. In some cases, the N-terminal domain binds directly to a signaling molecule. However, the N-terminal domain of most EBPs is modified (e.g., by phosphorylation) by a signal transduction partner such as a histidine protein kinase that detects the signal^11^. Because σ^54^-RNA polymerase requires the energy from EBP-catalyzed ATP hydrolysis to initiate transcription and the EBP’s ATPase activity is controlled by signal input, the σ^54^ system is able to tightly control transcription of its target genes.

Historically, σ^54^ was viewed as specialized regulatory system that was mainly dedicated to transcription of genes involved in nitrogen assimilation or nitrogen fixation^12,13^. In recent years however, it has become clear that the σ^54^ system is important for transcription of many types of bacterial genes. For example, the σ^54^ system in *Bacillus cereus*, *Pseudomonas aeruginosa* and *Escherichia coli* modulates transcription of genes involved amino acid metabolism, the response to reactive nitrogen species and the phage shock response, respectively^14–16^. In *Caulobacter crescentus*, *Pseudomonas aeruginosa*, *Pseudomonas putida* and *Vibrio cholerae* the σ^54^ system regulates genes that are important for flagellar biosynthesis and motility^17–21^, and in *Pseudomonas aeruginosa* the σ^54^ system is implicated in transcription of genes involved in quorum sensing, biofilm formation and virulence^22–24^.

The σ^54^ system in the soil bacterium *Myxococcus xanthus* has been studied extensively and has some rare properties. Namely, *M*. *xanthus* is one of the rare bacterial species in which the σ^54^ system has been linked to growth in nutrient rich conditions. For example, inactivation of the *nla4* or *nla18* EBP gene severely impairs *M*. *xanthus* growth in nutrient rich media^25–27^. Presumably, the relatively slow growth of the *nla4* mutant and *nla18* mutant is due at least in part to changes in the basal levels of the intracellular starvation signal (p)ppGpp and the resulting changes in gene expression.

*M. xanthus* also has an unusually large repertoire of 53 EBP genes^28^. All of the EBPs were characterized a number of years ago and many of the EBPs were implicated in motility^25,29,30^ and in starvation-induced biofilm formation^25,30–36^, which yields spore-filled aerial structures called fruiting bodies. Six of the EBPs that begin functioning in the early to middle stages of biofilm development, which is also known as fruiting body development, form a regulatory cascade^30^. This EBP cascade is reminiscent of the sigma factor cascade that controls the sequential stages of spore development in *Bacillus subtilis*^37^, as pairs of EBPs functioning at one stage of development directly activate transcription of an EBP gene important for the next developmental stage.

Nla28 is one of the early-functioning developmental EBPs that participates in the transcriptional cascade. A putative tandem repeat promoter binding site for Nla28 dimers was identified and analyzed using bioinformatics and experimentation^30,38,39^. The consensus Nla28 binding site [CT(C/G)CG(C/G)AG consensus half site], which was generated from these studies, was subsequently used to search the *M*. *xanthus* genome sequence for Nla28 target promoters/genes located outside the EBP cascade^28,39^. A number of these putative Nla28 target promoters were identified in natural product gene clusters. This was an intriguing finding, as Volz et al. previously showed that two *M*. *xanthus* EBPs (HsfA and MXAN4899) are capable of binding to fragments of natural product gene promoters^40^. Furthermore, it was previously suggested that the σ^54^ system might be a key regulator of polyketide (PK) and non-ribosomal peptide (NRP) natural product genes in *M*. *xanthus*, and in bacteria in general, based on bioinformatics^38^.

Here, we explored the idea that the σ^54^ system modulates expression of *M*. *xanthus* natural product genes. We focused on three potential σ^54^ promoter targets of Nla28 that are located in *M*. *xanthus* natural product gene clusters^38^. The activities of the three natural product promoters increase during growth, with peak activities occurring during the transition into stationary phase. The activities of the promoters also increase during starvation-induced development. These findings are consistent previous studies linking expression of bacterial natural product genes to changes in nutritional status^41–45^. Our in vivo and in vitro mutational analyses indicate that three natural product promoters are indeed σ^54^ promoter elements and direct targets of Nla28, as predicted via bioinformatics. An examination of the characterized and uncharacterized σ^54^ promoters in *M*. *xanthus* natural product gene clusters yielded a striking result; the vast majority are predicted to be intragenic not intergenic promoters. These results are consistent with previous experimental findings, which placed many characterized *M*. *xanthus* σ^54^ promoters in intragenic regions^30,39^.

## Materials and methods

### Bacterial strains, plasmids and media

Bacterial strains and plasmids used in the study are listed in Table S1. *M*. *xanthus* strains were grown at 32 °C in CTTYE broth [1% Casitone, 0.2% yeast extract, 10 mM Tris (pH 8.0), 1 mM KH_2_PO_4_ (pH 7.6), 8 mM MgSO_4_] or on CTTYE plates containing 1.5% agar. Fifty μg/ml of kanamycin or 10 μg/ml of tetracycline were added to CTTYE broth and CTTYE agar plates as needed. CTT soft agar (CTTSA), which is used to plate electroporated *M*. *xanthus* cells, contains 1% Casitone, 10 mM Tris (pH 8.0), 1.0 mM KH_2_PO_4_ (pH 7.6), 8.0 mM MgSO_4_, and 0.7% agar. Submerged culture development of *M*. *xanthus* strains occurred in 24-well polystyrene plates containing 100 μl of MC7 buffer [10 mM morpholinepropanesulfonic acid (MOPS; pH 7.0), 1 mM CaCl_2_]. Unless otherwise stated, *E*. *coli* strains were grown in Luria–Bertani (LB) broth [0.5% yeast extract, 1% tryptone, 1% NaCl) or on LB plates containing 1.5% agar. LB broth and LB plates were supplemented with 100 μg/ml of ampicillin, 50 μg/ml of kanamycin or 10 μg/ml of tetracycline as needed. For Nla28-DBD expression, *E*. *coli* strains were grown in rich LB broth [0.5% yeast extract, 1% tryptone, 0.5% NaCl, 0.2% glucose] supplemented with 100 μg/ml of ampicillin.

### *M*.* xanthus* growth and development

*M*. *xanthus* strains were grown by inoculating cells into flasks containing CTTYE broth and incubating the cultures at 32 °C with vigorous swirling. Development was induced as previously described^25^. Briefly, *M*. *xanthus* cells were grown in CTTYE broth until the cultures reached a density of approximately 5 × 10^8^ cells/ml, the cells were pelleted, the supernatant was removed, and the cells were resuspended in MC7 buffer to a density of 5 × 10^9^ cells/ml. Forty μl aliquots of the cell suspensions were placed into polystyrene plate wells containing 100 μl of MC7 buffer and the polystyrene plates were transferred to a 32 °C incubator for 24 h.

### Standard DNA procedures

Chromosomal DNA from wild-type *M. xanthus* strain DK1622^46^ was extracted using ZYMO Research gDNA extraction kit. Oligonucleotides used in PCR reactions were synthesized by Integrated DNA Technologies (IDT) and are listed in Table S2. Plasmid DNA was extracted using the Promega Nucleic acid purification kit. Amplified and digested DNA fragments were purified using the Gel Extraction Minipreps kit of Bio Basic. For all kits, the manufacturer’s protocols were used. The compositions of all plasmids and promoter fragments were confirmed by DNA sequencing (Genewiz).

### Site-directed mutations

Site-directed mutations in putative σ^54^ promoter elements were generated using the Quick Lightning Mutagenesis Kit from Agilent Technologies and the manufacturer’s protocol. Briefly, promoter fragments containing the putative σ^54^-RNA polymerase binding site in the − 12 and − 24-bp regions and the upstream Nla28 tandem repeat binding site were cloned into pCR 2.1 TOPO vector (Invitrogen). Mutations in the − 12-bp region, the − 24-bp region, the spacer between the − 12 and − 24-bp regions were generated using primers carrying the appropriate nucleotide changes (Table S2), plasmids containing the promoter fragments and PfuUltra DNA polymerase. Parental plasmid DNA was removed by digesting with DpnI and transformed into *E*. *coli* for conversion into duplex form. Plasmid-borne promoter mutations were verified by DNA sequence analysis. Promoter fragments carrying Nla28 binding site mutations (P_EM1286_, P_EM1579_ and P_EM3778_) were synthesized by IDT; the first putative Nla28 half binding site in each promoter was changed to AAAAAAAA. The mutant promoter fragments were then subcloned into the promoterless *lacZ* expression vector pREG1727^47^, introduced into *M*. *xanthus* strains and analyzed as described below.

### In vivo analysis of wild-type and mutant promoters

Wild-type and mutant MXAN1286, MXAN1579, MXAN1603 and MXAN3778 promoter fragments were cloned into the promoterless *lacZ* expression vector pREG1727 to create *lacZ* transcriptional fusions^47^. The plasmids were introduced into strain DK1622 or a derivative of strain DK1622 carrying an insertion in the *nla28* gene, and cells carrying a plasmid integrated at the Mx8 phage attachment site in the chromosome were identified via PCR. The in vivo activities of wild-type and mutant promoters were determined by measuring the specific activities of β-galactosidase in cells developing in submerged cultures for 1, 2, 6, 12 or 24 h, or growing in CTTYE broth for various amounts of time^48,49^. Mean wild-type and mutant promoter activities were compared using a two-way analysis of variance (ANOVA) and Tukey’s multiple comparisons post hoc tests, considering two independent variables in each group. The significance level was set at p < 0.05 or lower. GraphPad Prism software v8.4 (GraphPad Software, La Jolla, CA, USA) was used for all analyses.

### Expression and purification of Nla28-DBD

A fragment of the *nla28* gene corresponding to the Nla28 DNA binding domain (Nla28-DBD)^30^ was PCR amplified using gene-specific primers (Table S2), and then cloned into the pMAL-c5x vector. The resulting plasmid, which creates an N-terminal Maltose Binding Protein (MBP) fusion to Nla28-DBD, was introduced into *E*. *coli* strain BL21 (DE3) using chemical transformation. Cells containing the Nla28-DBD expression plasmids were grown in rich LB broth to a density of 2 × 10^8^ cells/ml. Protein expression was induced by the addition of 0.3 mM IPTG to the culture and the subsequent incubation of the culture for 12 h at 15 °C. Cells were pelleted via centrifugation and resuspended in 25 ml column buffer (20 mM Tris–HCl, 200 mM NaCl, 1 mM EDTA, 5 U/ml DNase I) per liter of culture. The resuspended cells were lysed by a combination of freeze-thawing and sonication, and pelleted by centrifugation. The crude extract (supernatant) containing Nla28-DBD was diluted by adding 125 ml of cold column buffer to every 25 ml aliquot of crude extract. For purification of Nla28-DBD from the diluted crude extract, 15 ml of amylose resin was placed in a 2.5 × 10 cm column and the amylose resin was washed with 250 ml cold column buffer. Diluted crude extract containing Nla28-DBD was loaded onto amylose columns at a flow rate of 5 ml/min and washed with 600 ml cold column buffer at a flow rate of 10 ml/min. Nla28-DBD was eluted using 100 ml cold column buffer containing 10 mM maltose; the flow rate was 5 ml/min and 20 fractions containing 5 ml were collected. The presence of eluted Nla28-DBD was detected by UV absorbance at 280 nm. Nla28-DBD- containing fractions were pooled and incubated with 1 mg of Factor Xa at 4 °C overnight to cleave the MBP tag. Nla28-DBD was separated from MBP and concentrated to about 1 mg/ml using Amicon Ultra centrifugal filter units (EMD Millipore). SDS-PAGE and Bradford assays were used to determinate the purity and concentration of Nla28-DBD.

### Electrophoretic mobility shift assays (EMSAs)

Purified Nla28-DBD was expected to bind to wild-type MXAN1286, MXAN1579 and MXAN3778 promoter fragments carrying a putative Nla28 tandem repeat binding site. Using the 5′ Cy5-labelled oligonucleotides shown in Table S2, Cy5-labelled MXAN1286, MXAN1579 and MXAN3778 promoter fragments (Cy5-P_1286_, Cy5-P_1579_ and Cy5-P_3778_) were generated via PCR; each promoter fragment contained a putative wild-type Nla28 binding site. Three mutant derivatives of these 5′ Cy5-labelled promoter fragments (Cy5-P_mut1286_, Cy5-P_mut1579_ and Cy5-P_mut3778_) were synthesized; the first putative Nla28 half binding site in each promoter was changed to AAAAAAAA. All PCR-generated and synthesized promoter fragments were gel-purified and used in subsequent EMSAs. In EMSA reactions, 2 μM purified Nla28-DBD was incubated with 1 ng of 5′ Cy5-labelled wild-type promoter fragment (Cy5-P_1286_, Cy5-P_1579_ and Cy5-P_3778_) or 5′ Cy5-labelled mutant promoter fragment (Cy5-P_mut1286_, Cy5-P_mut1579_ and Cy5-P_mut3778_) in EMSA buffer (25 mM Tris acetate, 8.0 mM magnesium acetate, 10 mM KCl, 1 mM DTT, pH 8.0) for 30 min at 30 °C. The samples were then analyzed using PAGE under non-denaturing conditions and imaged using a Bio-Rad imager.

## Results

### Identifying putative σ^54^ promoter elements in *M. xanthus* natural product gene clusters

In a previous study^38^, the algorithm developed by Studholme et al*.*^50^ was used to examine whether the σ^54^ system might be a common regulator of bacterial natural product genes. Namely, 180 annotated PK and NRP gene clusters from 58 bacterial species were analyzed for sequences that closely match the σ^54^ promoter consensus in the − 12-bp region and in the − 24-bp region (i.e., the regions of σ^54^-RNA polymerase binding). The results, which uncovered 124 clusters with at least one σ^54^ promoter based on consensus matching, supported the idea that the σ^54^ system might be a general regulator of bacterial natural product genes.

The goal of the work presented here was to examine whether a major producer of bacterial natural products (*M*. *xanthus*) uses the σ^54^ system for transcription of PK and NRP gene clusters, as predicted in the bioinformatics analysis of Stevens et al*.*^38^. We focused on the putative σ^54^ promoters upstream of MXAN1286, MXAN1579, MXAN1603 and MXAN3778, as these genes were linked to natural product biosynthetic genes/clusters. Namely, the MXAN1286 gene is embedded in a putative NRP gene cluster and flanked by genes that are likely to be involved in NRP biosynthesis. MXAN1579 and MXAN1603 are predicted to be part of another *M*. *xanthus* NRP gene cluster. MXAN1603 is a putative non-ribosomal peptide synthetase gene. MXAN1579 is embedded in the cluster and flanked by genes such as MXAN1603 that are likely to be involved in NRP biosynthesis. MXAN3778 is adjacent to a putative hybrid non-ribosomal peptide synthetase/type I polyketide synthase gene and possibly part of the same operon^38^.

**Figure 1:**
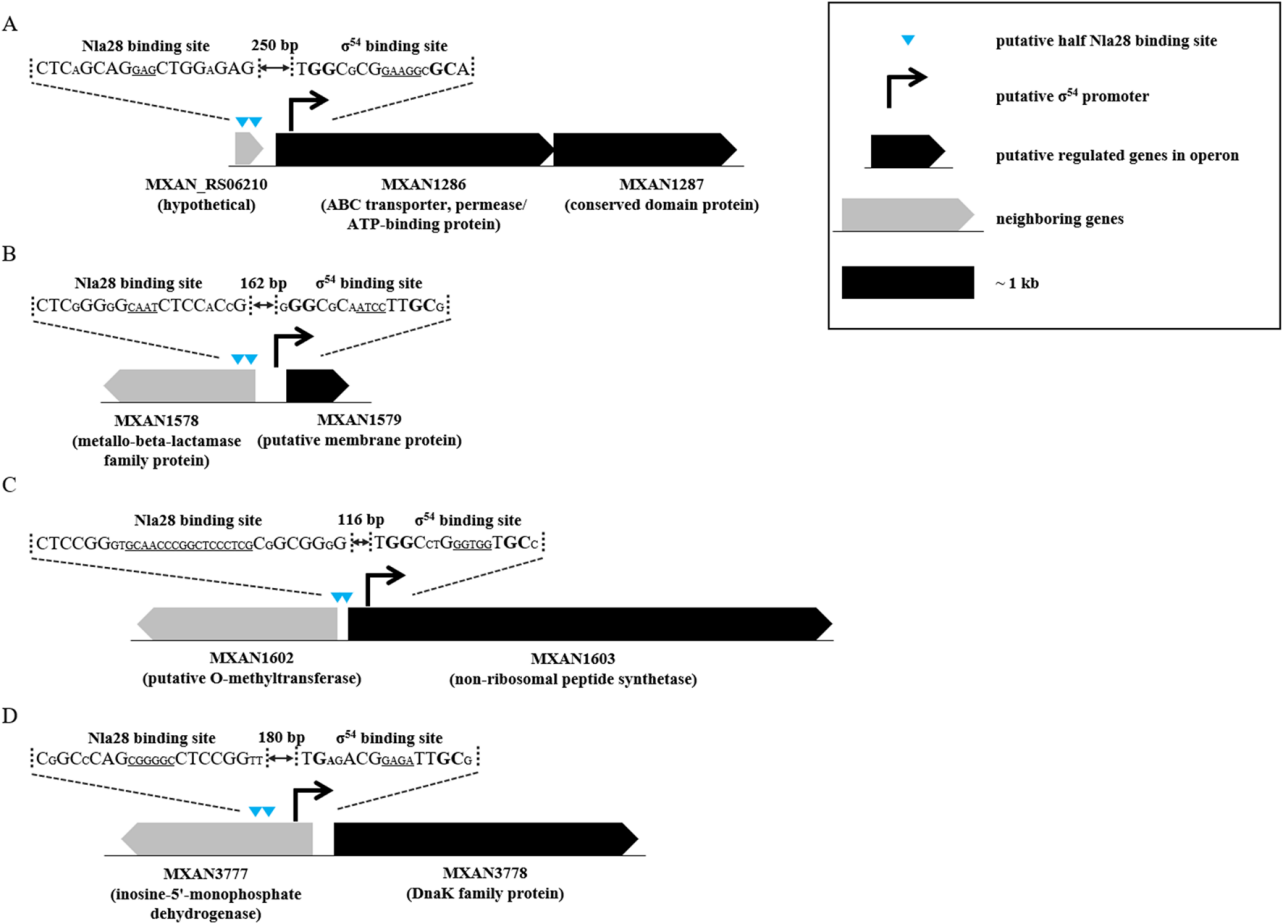
The promoter regions of the MXAN1286, MXAN1579, MXAN1603 and MXAN3778 natural product loci. Nucleotides the match those in the consensus Nla28 binding site or the consensus σ^54^ RNA polymerase binding site are relatively large. The conserved GC dinucleotide in − 12-bp region and the conserved GG dinucleotide in − 24-bp region of the putative σ^54^ RNA polymerase binding sites are in bold. The underlined nucleotides represent the spacers between the two half Nla28 binding sites or the spacers between − 12 and − 24-bp promoter regions.

**Figure 2:**
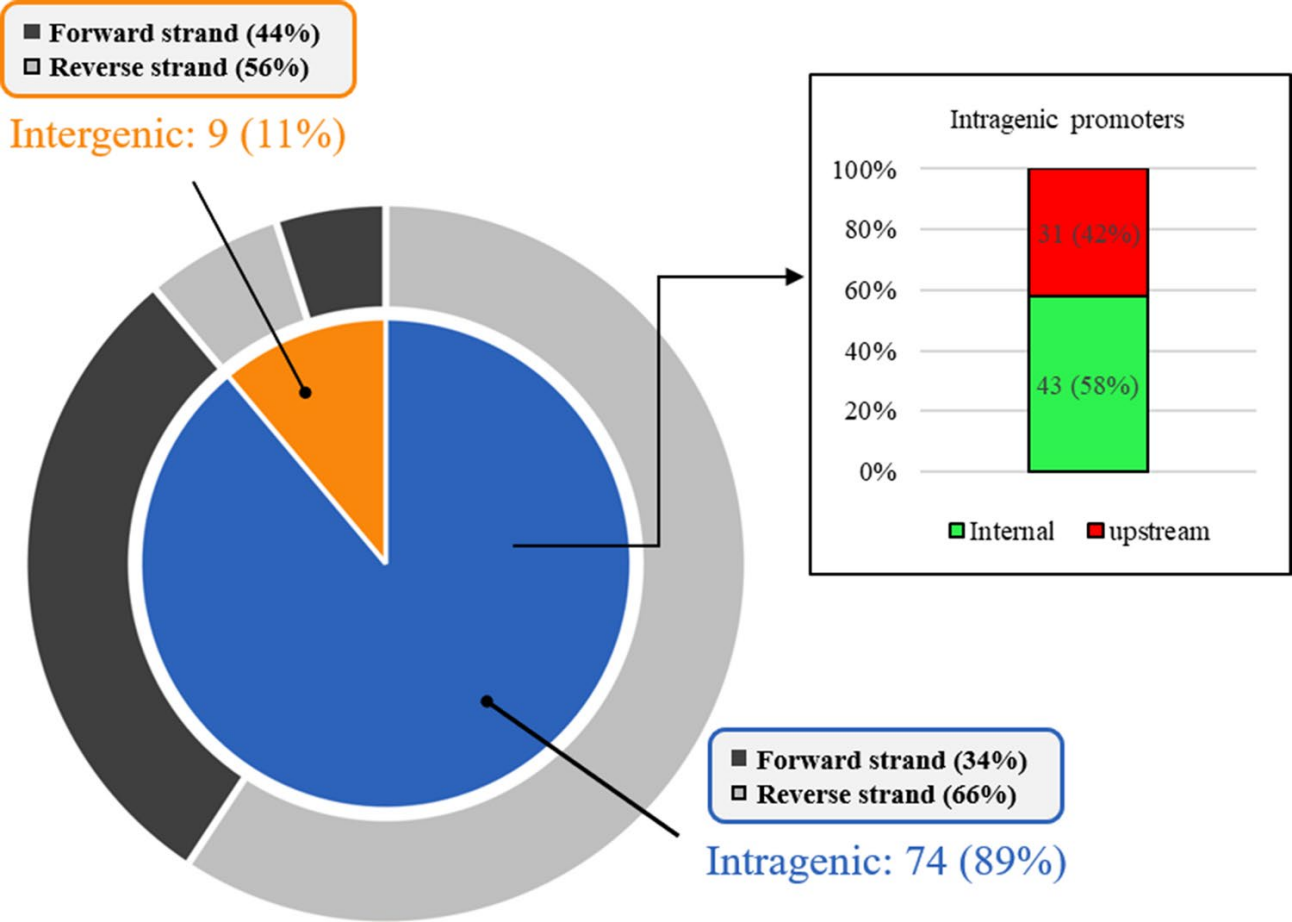
Locations of putative PK/NRP σ^54^ promoters identified in the *M. xanthus* genome. Of the 83 putative PK/NRP σ^54^ promoters identified in *M. xanthus* genome sequence, 74 (89%) are located in protein coding sequences (intragenic promoters) and 9 (11%) are located in non-coding sequences (intergenic promoters). Of the 74 intragenic promoters, 43 are located within a protein coding sequence in an operon or within the protein coding sequence of a single gene (internal promoters), and 31 are located in the protein coding sequence of an upstream gene (upstream promoters).

The MXAN1286, MXAN1579, MXAN1603 and MXAN3778 genes were also identified as potential targets of the EBP Nla28^30,39^; 8-bp repeat sequences, which are close matches to the consensus Nla28 half binding site, were identified upstream of the putative − 12 and − 24-bp regions (Fig. 1). It is notable that six of the eight putative Nla28 half binding sites and three of the four putative σ^54^-RNA polymerase binding sites are located within protein coding sequences. Indeed, residence in an intragenic region is common among the putative PK and NRP σ^54^ promoters identified in the *M*. *xanthus* genome (Fig. 2, Table S3 and Figure S1), and among the σ^54^ promoters known to be regulated by the EBP Nla28^30,39^. It is also noteworthy that many of the putative σ^54^ promoter elements are located within operons; they might serve as internal promoters (Fig. 2, Table S3 and Figure S1).

### Mutations in the putative − 12-bp region, − 24-bp region or spacer region impair the in vivo activities of natural product promoters

σ^54^ promoters typically have a GC dinucleotide in the − 12-bp region and a GG dinucleotide in the − 24-bp region^1,2^. These dinucleotides and the 4-bp spacer between the − 12 and − 24-bp regions are often referred to as the hallmarks of σ^54^ promoters. Indeed, the putative σ^54^ promoters in the MXAN1286, MXAN1579 and MXAN1603 natural product loci appear to have these hallmarks (Fig. 1). As for the σ^54^ promoter identified in the MXAN3778 locus, one hallmark variation is apparent. Namely, the − 24-bp region has GA instead of a GG dinucleotide. Despite this variation in − 24-bp region dinucleotide, MXAN3778 was classified as a potential σ^54^ promoter, as a 1-bp change in either the GC or GG dinucleotide has been identified in a number of characterized σ^54^ promoters, including the σ^54^ promoters in the *M*. *xanthus asgE*, *spi* and *nla6* loci^30,51–53^.

To confirm that the natural product loci have bona fide σ^54^ promoter elements, we analyzed the putative σ^54^ promoter hallmarks via mutational analysis. In particular, a 446-bp DNA fragment of the MXAN1286 promoter region, a 402-bp fragment of the MXAN1579 promoter region, a 518 bp fragment of the MXAN1603 promoter region and a 600-bp fragment of the MXAN3778 promoter region were used to generate mutations in the putative − 12-bp, − 24-bp and spacer regions. We should note that the MXAN1286 promoter fragment contains both of the putative σ^54^ promoters located in the 5′ end of the MXAN1286 gene and the MXAN1579 promoter fragment contains the two putative intergenic σ^54^ promoters that are oriented in the appropriate direction with respect to MXAN1579 transcription (Table S3 and Figure S1). However, we focused the mutational analysis on the putative σ^54^ promoter that is the farthest downstream in the MXAN1286 coding sequence (+ 111 bp; see Table S3) and the putative σ^54^ promoter that is farthest upstream of the predicted transcriptional start site of MXAN1579 gene (− 104 bp; see Table S3) after considering a number of factors, including the presence of σ^54^ promoter hallmarks, the overall match to the σ^54^ promoter consensus sequence [TGGCACG-4 N-TTGC(T/A)] and the distance from putative Nla28 binding sites^1,2^. The mutations that we generated are the following: the GC dinucleotide in the − 12-bp region was replaced with a TT, the GG (GA in MXAN3778) dinucleotide in the − 24-bp region was replaced with a TT, or 1 bp in the spacer between the − 12 and − 24-bp regions was deleted. Subsequently, wild-type and mutant promoter fragments were fused to the promoterless *lacZ* gene in plasmid pREG1727^47^ and the *lacZ* transcriptional fusion plasmids were introduced into wild-type *M. xanthus* strain DK1622 (the plasmids integrated at the Mx8 phage attachment site in the chromosome).

**Figure 3:**
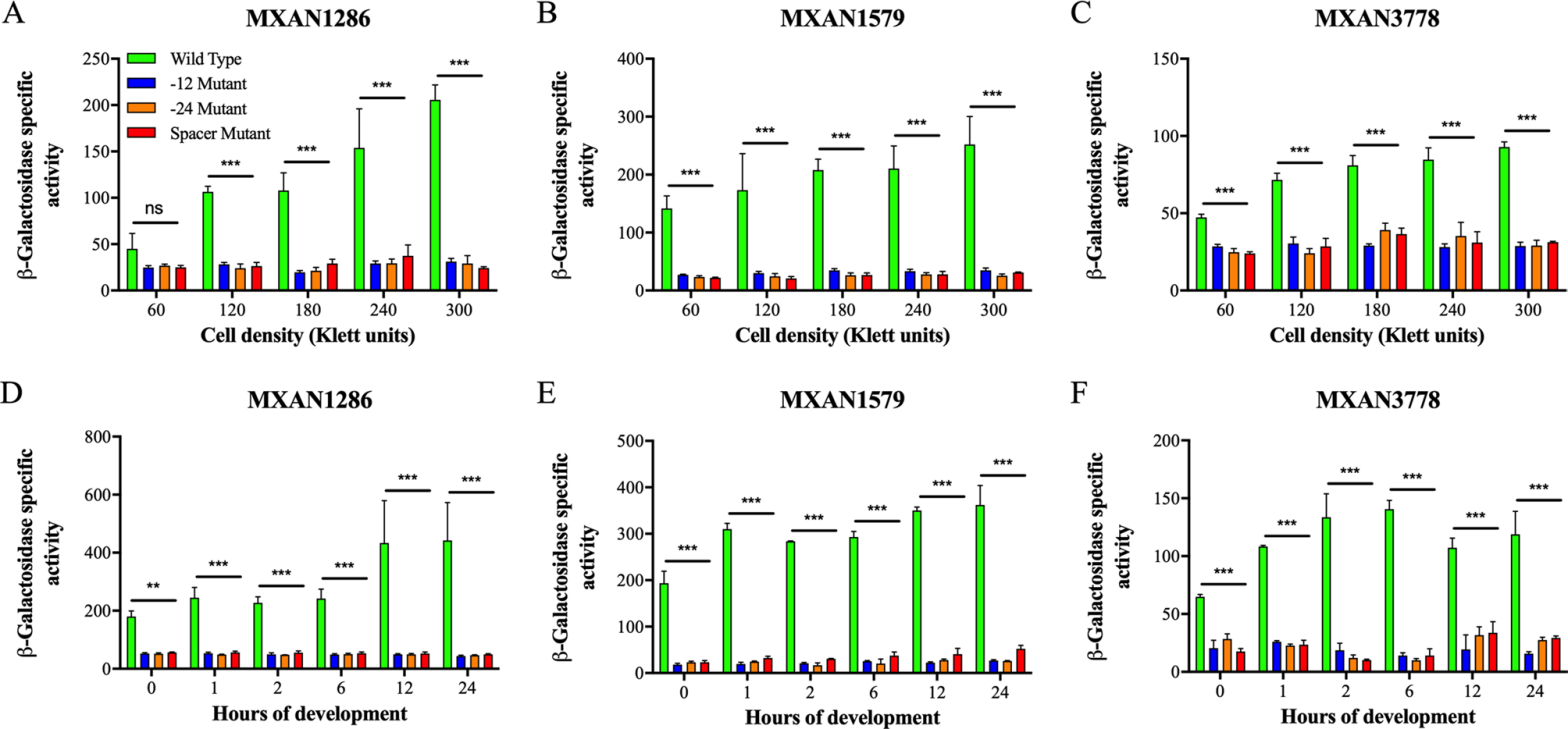
In vivo activities of wild-type MXAN1286, MXAN1579 and MXAN3778 promoters and derivatives of the promoters carrying a mutation in the putative − 12-bp region, − 24-bp region or spacer region. Wild-type and mutant fragments of the MXAN1286, MXAN1579 and MXAN3778 promoters were cloned into a *lacZ* expression vector and transferred to the wild-type *M. xanthus* strain DK1622. At various cell densities during growth (**A**–**C**) and time points during development (**D**–**F**), β-galactosidase-specific activities (defined as nanomoles of ONP produced per minute per milligram of protein) in cells carrying a wild-type or a mutant promoter fragment were determined. (N = 3 at each density or time point; error bars are standard deviations of the means; ***p < 0.001; **p < 0.01; *p < 0.05 for in vivo activities of mutant promoters versus wild-type promoters).

Wild-type and mutant promoter activities during growth in CTTYE broth and fruiting body development in MC7 starvation buffer were inferred from the levels of *lacZ* expression. As shown in Fig. 3A–C, the in vivo activities of the MXAN1286, MXAN1579 and MXAN3778 promoters increased about 4.6-fold, 1.8-fold and twofold, respectively, during growth in CTTYE broth. It is notable that peak levels of promoter activity occurred at the highest cell densities, which correspond to the transition into stationary phase and presumably nutrient depletion. This of course agrees with the data shown in Fig. 3D–F, which revealed a 1.8- to 2.5-fold increase in the vivo activities of the three promoters during development in MC7 starvation buffer. Mutations in the − 12-bp region, the − 24-bp region and spacer dramatically reduced (about 3.2- to 11.1-fold) the activities of these promoters at all cell densities during growth and time points in development. Thus, mutations in the putative sites of σ^54-^RNA polymerase binding substantially impacted the activities of the MXAN1286, MXAN1579 and MXAN3778 promoters in growing and developing cells, supporting the prediction that the three natural product loci use σ^54^ promoter elements for transcription.

In contrast to the other promoters, the MXAN1603 promoter only showed a slight increase (about 1.3-fold) in activity during growth in CTTYE (Figure S2A) and during development in MC7 starvation buffer (Figure S2B). Furthermore, mutations in the − 12-bp region, the − 24-bp region and the spacer caused a modest, but statistically significant decrease (P < 0.001) in promoter activity at all cell densities during growth and after 1 h of development (Figure S2). Thus, mutations in the putative σ^54^-RNA polymerase binding site in the MXAN1603 promoter region only had a modest impact on growth-related and developmental activities. Our interpretation of this result is that the fragment of the MXAN1603 promoter region contains an unidentified promoter element that is responsible for the majority of the observed development and growth-related activities and that the putative σ^54^-type promoter element only makes a minor contribution to the observed activities.

### The in vivo activities of natural product promoters are impacted by inactivation of the *nla28* gene

**Figure 4:**
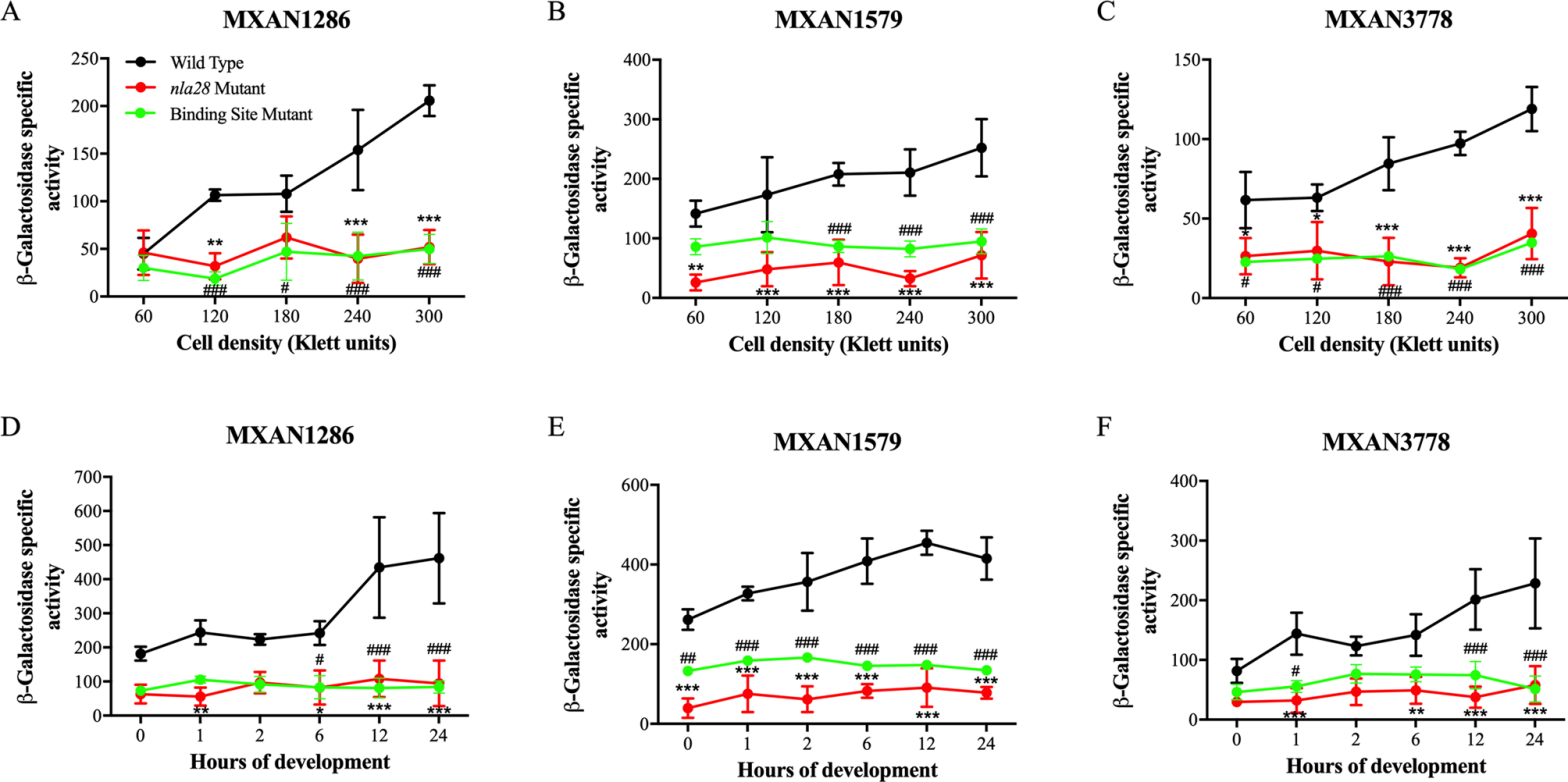
In vivo activities of MXAN1286, MXAN1579 and MXAN3778 promoters in *nla28* mutant cells and when Nla28 binding sites are mutated. Wild-type fragments of the MXAN1286, MXAN1579 and MXAN3778 promoters were cloned into a *lacZ* expression vector and transferred to the wild-type *M. xanthus* strain DK1622 (Wild Type) or to a derivative of strain DK1622 with an inactivated *nla28* gene (*nla28* Mutant). In addition, fragments of the MXAN1286, MXAN1579 and MXAN3778 promoters containing one mutated Nla28 half binding site were cloned into a *lacZ* expression vector and transferred to strain DK1622 (Binding Site Mutant). At various cell densities during growth (**A**–**C**) and time points during development (**D**–**F**), β-galactosidase-specific activities in cells carrying a wild-type or a mutant promoter fragment were determined. (N = 3 at each density or time point; error bars are standard deviations of the means; ***p < 0.001; **p < 0.01; *p < 0.05 for in vivo activities of wild-type promoters in *nla28* mutant versus in wild-type cells; ^###^p < 0.001; ^##^p < 0.01; ^#^p < 0.05 for in vivo activities of binding site mutant promoters versus wild-type promoters in wild-type cells).

EBPs are essential for transcription at σ^54^ promoters, as EBP-mediated ATP hydrolysis opens the σ^54^-RNA polymerase promoter complex so that transcription can initiate^3–5^. Since the σ^54^ promoters in the MXAN1286, MXAN1579, and MXAN3778 loci were identified as potential targets of Nla28, we determined whether the activities of wild-type promoter fragments are reduced as predicted in a mutant containing an inactivated *nla28* gene^25^ (Note that the MXAN1603 σ^54^ promoter was not analyzed further because it is unlikely to be the primary promoter used during growth or development). MXAN1286, MXAN1579, and MXAN3778 promoter activities in wild-type and *nla28* mutant cells grown in CTTYE broth are shown in Fig. 4A–C. As predicted, inactivation of *nla28* abolished the growth phase regulation of all three promoters and caused about 3.1- to fourfold reduction in peak promoter activities at the highest cell densities. Inactivation of *nla28* also abolished the developmental activities of the promoters, as the promoters did not show the typical increases in activities when *nla28* cells were placed in MC7 starvation buffer (Fig. 4D–F). Indeed, the peak developmental promoter activities in *nla28* mutant cells were reduced about 3.4- to 5.0-fold relative to the corresponding peak activities in wild-type cells. These findings indicate that Nla28 is crucial for the observed growth-related and developmental activities of the MXAN1286, MXAN1579, and MXAN3778 natural product promoters.

### Mutations in putative Nla28 half sites impact the in vivo activities of natural product promoters

As noted above, we identified 8-bp repeat sequences, which are close matches to the consensus Nla28 half binding site [CT(C/G)CG(C/G)AG], in the σ^54^ promoters under study here (see Fig. 1). To examine whether the σ^54^ promoters are directly regulated by Nla28 and to further confirm that the promoters are members of the σ^54^ family, mutations were generated in the putative Nla28 binding sites in the MXAN1286, MXAN1579, and MXAN3778 promoter fragments noted above. Namely, the distal (relative to the − 12 and − 24-bp regions) Nla28 half binding site in each promoter fragment was converted to all A nucleotides. Wild-type and mutant promoters were introduced into wild-type strain DK1622 and promoter activities during growth in CTTYE broth and development in in MC7 starvation buffer were determined (Fig. 4). The data revealed that Nla28 binding site mutations abolish the growth phase regulation of all three promoters (Fig. 4A–C). Indeed, the peak mutant promoter activities, which were observed at the highest cell density, were reduced about 2.6- to fivefold compared to that of the corresponding wild-type promoter. Similarly, wild-type MXAN1286, MXAN1579, and MXAN3778 promoters showed increased activities during development and the Nla28 binding site mutations abolished this developmental regulation (Fig. 4D–F). Furthermore, the peak developmental activities of the mutant promoters were reduced from about 2.8- to 4.8-fold. These findings are consistent with the idea that Nla28 directly regulates the MXAN1286, MXAN1579, and MXAN3778 σ^54^ promoters, that the 8-bp repeats that we identified are Nla28 binding sites and that Nla28 is crucial for growth-related and developmental promoter activities.

### In vitro analyses indicate that purified Nla28-DBD binds to natural product promoter fragments carrying a wild-type Nla28 binding site, but not to fragments carrying a mutated Nla28 binding site

**Figure 5:**
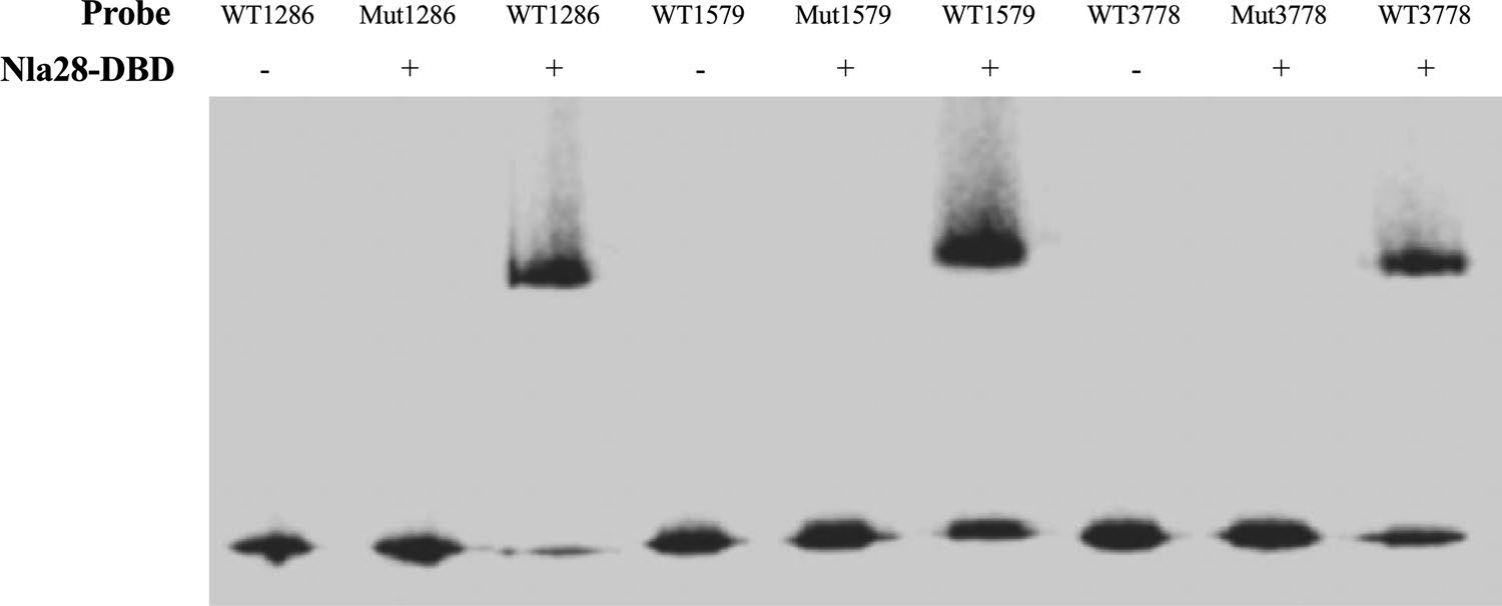
EMSAs performed with Nla28-DBD and a MXAN1286, MXAN1579 or MXAN3778 promoter fragment carrying a wild-type or mutated Nla28 binding site. Binding reactions were performed with (+) or without (−) 2 μM of purified Nla28-DBD and a Cy5 end-labeled promoter fragment containing a wild-type (WT) or mutated (Mut) Nla28 binding site.

Electrophoretic mobility shift assays (EMSAs) were used to confirm that the MXAN1286, MXAN1579, and MXAN3778 natural product promoters are targets of the Nla28 EBP. In particular, we used EMSAs to determine whether the purified DNA binding domain of Nla28 (Nla28-DBD) is capable of binding a fragment of the MXAN1286 promoter, MXAN1579 promoter, and MXAN3778 promoter. Each promoter fragment, which corresponded to DNA upstream of − 12 and − 24-bp regions, contained a putative binding site for a Nla28 dimer. As shown in Fig. 5, Nla28-DBD is capable of binding to a MXAN1286, MXAN1579 and MXAN3778 promoter fragment that has a wild-type Nla28 binding site. However, when the distal Nla28 half binding site in each promoter fragment was converted to all A nucleotides, no Nla28-DBD binding was detected (Fig. 5). These findings provide further support that the Nla28 EBP directly regulates the σ^54^ promoter elements of the MXAN1286, MXAN1579 and MXAN3778 natural product loci and that the tandem repeats that we identified in the σ^54^ promoter elements are Nla28 binding sites.

## Discussion

For decades, bacterial-derived PK and NRP natural products have been a crucial source of therapeutic agents such as antibiotics and yet little information about the regulation of these genes has been uncovered. In a notable study in 2012, Volz et al. showed that two *M*. *xanthus* EBPs (HsfA and MXAN4899) are capable of binding to fragments of natural product gene promoters^40^. With this information and the preliminary data from Nla28 studies in mind, Stevens et al. asked if the σ^54^ system might be a common regulator natural product genes^38^. Namely, a bioinformatics analysis was used to search for putative σ^54^ promoters in the sequences of 180 PK or NRP gene clusters from 58 bacterial species. The results, which revealed that about 70% of natural product gene clusters have at least one putative σ^54^ promoter, suggested that the σ^54^ system might indeed be a common regulator of natural product genes.

One of the goals of this study was to analyze the bioinformatics data experimentally and the putative natural product promoter targets of Nla28 seemed particularly well suited for such a study, given our knowledge of Nla28-mediated regulation. Furthermore, *M*. *xanthus* is an excellent system to study natural product gene regulation, as this bacterium is a major producer of PKs and NRPs and over 80 putative σ^54^ promoters were identified in the PK and NRP gene clusters of strain DK1622^38^ (Table S3 and Figure S1).

Our in vivo and in vitro data indicate that three of the *M*. *xanthus* natural product promoter regions characterized here (MXAN1579, MXAN3778 and MXAN1286) contain bona fide σ^54^ promoter elements and that the σ^54^ promoters are targets of the EBP Nla28. Our results also indicate that the σ^54^ promoters are crucial for both growth-related and developmental activities. We propose that the fourth natural product promoter region that we characterized, MXAN1603, also contains a σ^54^ promoter. However, it seems likely that the MXAN1603 promoter region contains an unidentified promoter element that is responsible for the majority of the observed developmental and growth-related activities and the σ^54^-type promoter only makes a minor contribution to these activities.

Previous work indicated that the Nla28 EBP is a response regulator that forms a two component signal transduction system with the membrane-bound sensor histidine kinase Nla28S^54,55^. Nla28 begins modulating gene expression in the early stages of starvation-induced fruiting body development^25,30,39^, which led to the suggestion that the Nla28/Nla28S signal transduction system might be a general regulator of starvation-induced or stress-responsive genes^55^. The findings presented here are consistent with this idea. In particular, the MXAN1286, MXAN1579, and MXAN3778 σ^54^ promoters are induced in the early-middle stages of fruiting body development and the developmental activities of these promoters are dependent on Nla28 (Fig. 4D–F). The activities of the three promoters also increase during growth, with peaks corresponding to the transition into stationary phase and presumably nutrient depletion. As shown in Fig. 4A–C, this growth-phase regulation is abolished when Nla28 is absent, further linking the Nla28/Nla28S signal transduction system to the starvation response.

The above results point to nutrient depletion/starvation as the potential trigger for Nla28/Nla28S-mediated activation of the MXAN1286, MXAN1579 and MXAN3778 promoters. We suggest that the stringent response and accumulation of (p)ppGpp might also be a key inducing factor, as accumulation of this starvation signal is known to initiate *M*. *xanthus* fruiting body development^56,57^. Furthermore, it is reasonable to speculate that (p)ppGpp accumulates when *M*. *xanthus* transitions from exponential growth to stationary phase, since this seems to be the case in other bacterial species^58^. In addition, bacterial natural product gene expression was previously linked to (p)ppGpp accumulation^59^ and stringent response-associated genes such as *relA* and *spoT* have been linked to synthesis of bacterial natural products^41,42,60^.

It is interesting that three of the four natural product σ^54^ promoters that we characterized are located within the coding sequences of genes (intragenic) and not in intergenic regions (Fig. 1). In the MXAN1286 and MXAN1603 loci, the σ^54^ promoters are located in the coding sequence of the first gene of an operon and in the 5′ end of a single gene, respectively. In the other case (MXAN3778), the σ^54^ promoter is located in the coding sequence of an upstream gene (Fig. 1). With the exception of MXAN1603, the Nla28 binding sites of the natural product promoters are also intragenic, located in the coding sequence of an upstream gene (Fig. 1). These findings are counter to the commonly held view that bacterial promoter elements are typically located in intergenic regions^61–63^, but are supported by additional pieces of bioinformatic and experimental data. First, the vast majority of the putative natural product σ^54^ promoters shown in Figure S1 and listed in Table S3 are intragenic. Secondly, the majority of the characterized σ^54^ promoter targets of the *M*. *xanthus* EBP Nla6 are intragenic, located in the coding sequence of an upstream gene^53^. Thirdly, the vast majority of the developmental σ^54^ promoter targets of Nla28, which we recently characterized, are located in the coding sequence of an upstream gene or appear to be internal promoters in operons^30,39^. Lastly, genomic-wide studies in *Escherichia coli* and *Salmonella enterica* serovar *Typhimurium* uncovered an abundance of potential intragenic σ^54^ promoters and a number of these promoters were subsequently confirmed experimentally^64,65^. Together, these results suggest that σ^54^ promoter elements might indeed be commonly located in intragenic regions.

Many of the natural product σ^54^ promoters shown in Figure S1 and listed in Table S3 appear to be internal promoters; promoters located within a gene in an operon instead of upstream of the first gene in the operon. There are examples of internal promoters oriented in the antisense direction relative to the genes in an operon^65–68^; however, this does not seem to be the case for the above internal σ^54^ promoters. Instead, each promoter is predicted to yield mRNA corresponding to a subset of an operon’s genes. As noted in a recent review, one potential advantage of using internal promoters would be the ability to express only the genes needed in a particular environment^69^.

A second and relatively large group of putative natural product σ^54^ promoters is apparent from scanning Figure S1 and Table S3. Namely, many of the putative σ^54^ promoters are located in the coding sequence of a gene that is upstream of the single gene or operon they are predicted to regulate, even though the single gene or operon has an upstream and adjacent intergenic region that is long enough to accommodate a σ^54^ promoter (σ^54^-RNA polymerase binding site). The question that arises is, why would a σ^54^ promoter be located so far upstream of the single gene or operon whose transcription it aims to regulate? One possibility is that the natural product σ^54^ promoter is positioned at a distant location to produce a relatively long 5′ untranslated region (UTR) in the mRNA and that the 5′ UTR provides an additional layer of regulation for natural product gene expression. We should note that a substantial number of the internal σ^54^ promoters are predicted to yield relatively long 5′ UTRs as well. Why would many natural product genes need multiple levels of regulation? Natural product synthesis is often energy expensive and many natural products are toxic at high levels; hence, tight and signal-responsive regulation of natural product gene expression might be important in many cases.

Of course, addressing questions about the role and mechanism of σ^54^-mediated regulation of natural product genes and other types of bacterial genes will require researchers to go beyond predicting where σ^54^ promoters are located. Indeed, we argue that the work of confirming putative σ^54^ promoters and analyzing the mRNAs under σ^54^ control is crucial, as the results will help address a number of long-held assumptions about bacterial promoters and bacterial gene regulation in general.

## Supplementary Information

Supplementary Information

## Abbreviations

EBP: Enhancer binding protein
DBD: DNA binding domain
PK: Polyketide
NRP: Non-ribosomal peptide
UTR: Untranslated region
