# Supplementary material for: The σ54 system directly regulates bacterial natural product genes
Source: Sci Rep. 2021 Feb 26;11:4771. doi: 10.1038/s41598-021-84057-4 (PMC7910581; doi:10.1038/s41598-021-84057-4)
Supplement: Supplementary file 1 — Supplementary Information [file 41598_2021_84057_MOESM1_ESM.docx]

**The σ^54^ System Directly Regulates Bacterial Natural Product Genes**

Muqing Ma, Roy D. Welch and Anthony G. Garza*

Department of Biology, Syracuse University, Syracuse, NY 13244

*Corresponding author: Anthony Garza, Department of Biology, Syracuse University,

107 College Place, Syracuse, NY 13244, Telephone: 315-443-4746, E-mail: [agarza@syr.edu](mailto:agarza@syr.edu)

**Supplementary Table S1.** Bacterial strains and plasmids used in the current study

| **Bacterial Strains**  **or plasmid** | **Relevant characteristics** | **Source or reference** |
| --- | --- | --- |
| **Strains** |  |  |
| ***M. xanthus*** |  |  |
| DK1622 | Wild type | 46 |
| MM201 | Δ*nla28* derivative of DK1622 | This study |
| ***E. coli*** |  |  |
| Top 10 | Cloning host;  F^-^ *mcrA* Δ(*mrr-hsdRMS-mcrBC*) *φ80lacZ*ΔM15  Δ*lacX74 nupG recA1 ara*Δ*139* Δ(*ara-leu*)*7697*  *galE15 galK16 rpsL* (Str^R^) *endA1* λ^-^ | Invitrogen |
| BL21(DE3) | Nla28-DBD protein expression host;  *fhuA2 [lon] ompT gal* (*λ*DE3) [*dcm*] *ΔhsdS* | New England Biolabs |
| **Plasmids** |  |  |
| pCR 2.1 TOPO | Cloning vector; Km^r^ | Invitrogen |
| pREG1727 | Vector containing promoterless *lacZ*; Km^r^ | 47 |
| pSWU22 | Vector used for *nla28* inactivation; Tet^r^ | 25 |
| pMAL-c5x | Maltose-binding protein (MBP) fusion vector for Nla28-DBD protein expression; Amp^r^ | New England Biolabs |
| pMM101 | WT MXAN1286 promoter in pREG1727; Km^r^ | This study |
| pMM101a | -12 region mutant MXAN1286 promoter in pREG1727; Km^r^ | This study |
| pMM101b | -24 region mutant MXAN1286 promoter in pREG1727; Km^r^ | This study |
| pMM101c | -12/24 spacer mutant MXAN1286 promoter in pREG1727; Km^r^ | This study |
| pMM101d | Half Nla28 EBP binding site mutant MXAN1286 promoter in pREG1727; Km^r^ | This study |
|  |  |  |
| pMM102 | WT MXAN1579 promoter in pREG1727; Km^r^ | This study |
| pMM102a | -12 region mutated MXAN1579 promoter in pREG1727; Km^r^ | This study |
| pMM102b | -24 region mutated MXAN1579 promoter in pREG1727; Km^r^ | This study |
| pMM102c | -12/24 spacer mutated MXAN1579 promoter in pREG1727; Km^r^ | This study |
| pMM102d | Half Nla28 EBP binding site mutated MXAN1579 promoter in pREG1727; Km^r^ | This study |
| pMM103 | WT MXAN1603 promoter in pREG1727; Km^r^ | This study |
| pMM103a | -12 region mutated MXAN1603 promoter in pREG1727; Km^r^ | This study |
| pMM103b | -24 region mutated MXAN1603 promoter in pREG1727; Km^r^ | This study |
| pMM103c | -12/24 spacer mutated MXAN1603 promoter in pREG1727; Km^r^ | This study |
| pMM104 | WT MXAN3778 promoter in pREG1727; Km^r^ | This study |
| pMM104a | -12 region mutated MXAN3778 promoter in pREG1727; Km^r^ | This study |
| pMM104b | -24 region mutated MXAN3778 promoter in pREG1727; Km^r^ | This study |
| pMM104c | -12/24 spacer mutated MXAN3778 promoter in pREG1727; Km^r^ | This study |
| pMM104d | Half Nla28 EBP binding site mutated MXAN3778 promoter in pREG1727; Km^r^ | This study |
| pMM301 | 600-bp internal fragment of *nla28* gene in pSWU22 used for *nla28* inactivation; Tet^r^ | This study |
| pMM302 | Fragment for Nla28-DBD expression in pMAL-c5x; Amp^r^ | This study |

**Supplementary Table S2.** Oligonucleotides used in the current study

| **Application of oligonucleotides** | **Name** | **Sequence** |
| --- | --- | --- |
| **Amplification of WT σ^54^ promoter fragments** |  |  |
| MXAN1286 | MM101F | 5’ gaaagcttaacgccgctcgcaagg 3’ |
|  | MM101R | 5’ atcctagggaggtcggcgatgtg 3’ |
| MXAN1579 | MM102F | 5’ gcaagcttggggttggcgtagaagat 3’  v |
|  | MM102R | 5’ atcctagggaggaacaacccccg 3’ |
| MXAN1603 | MM103F | 5’ ataagcttgacgggctcgtgggg 3’ |
|  | MM103R | 5’ cacctagggcctcccgcatcaa 3’ |
| MXAN3778 | MM104F | 5’ aagctttcaccaccgggatgccgg 3’ |
|  | MM104R | 5’ cctagaagggcagcttcacgacgagc 3’ |
| **2-bp mutation in -12 region of σ^54^ promoter fragments** |  |  |
| MXAN1286 | MM101aF | 5' cgcaggtagagctgaaccttccgcgccaac 3' |
|  | MM101aR | 5' gttggcgcggaaggcttagctctacctgcg 3' |
| MXAN1579 | MM102aF | 5' ccacccgcgcaaaaggattgcgccccatccc 3' |
|  | MM102aR | 5' gggatggggcgcaatccttttgcgcgggtgg 3' |
| MXAN1603 | MM103aF | 5' ccgcgcgccgagaaaccacccaggcc 3' |
|  | MM103aR | 5' ggcctgggtggtttctcggcgcgcgg 3' |
| MXAN3778 | MM104aF | 5' gacgacccggctcactcaaaatctccgtctcaacatc 3' |
|  | MM104aR | 5' gatgttgagacggagattttgagtgagccgggtcgtc 3' |
| **2-bp mutation in -24 region of σ^54^ promoter fragments** |  |  |
| MXAN1286 | MM101bF | 5' gccttccgcgaaaacgcgcgattccggtgga 3' |
|  | MM101bR | 5' tccaccggaatcgcgcgttttcgcggaaggc 3' |
| MXAN1579 | MM102bF | 5' cgcaaggattgcgaaccatccccgcccg 3' |
|  | MM102bR | 5' cgggcggggatggttcgcaatccttgcg 3' |
| MXAN1603 | MM103bF | 5' accacccaggaaacttcgggcgacacccaga 3' |
|  | MM103bR | 5' tctgggtgtcgcccgaagtttcctgggtggt 3' |
| MXAN3778 | MM104bF | 5' cgcaatctccgtcaaaacatccccctcctgtcg 3' |
|  | MM104bR | 5' cgacaggagggggatgttttgacggagattgcg 3' |
| **1-bp deletion in the spacer region of σ^54^ promoter fragments** |  |  |
| MXAN1286 | MM101bF | 5' gcgttggcgcggaaagcttaacgccg 3' |
|  | MM101bR | 5' cggcgttaagctttccgcgccaacgc 3' |
| MXAN1579 | MM102bF | 5' gggatggggcgcaataagcttggggttg 3' |
|  | MM102bR | 5' caaccccaagcttattgcgccccatccc 3' |
| MXAN1603 | MM103bF | 5' gaagtggcctgggaagcttgacgggc 3' |
|  | MM103bR | 5' gcccgtcaagcttcccaggccacttc 3' |
| MXAN3778 | MM104bF | 5' gggatgttgagacgggattgcgagtgagcc 3' |
|  | MM104bR | 5' ggctcactcgcaatcccgtctcaacatccc 3' |
| **Amplification of 5’-labelled Cy5 WT σ^54^ promoter fragment for EMSAs** |  |  |
| Cy5-P_1286_ | MM109F | 5’ Cy5-gctcgtgtcgcgtccctacatcca 3’ |
|  | MM109R | 5’ acagggacagcgtccacacgacc 3’ |
| Cy5-P_1579_ | MM110F | 5’ Cy5-gcgtcttcttgtccgtaccgcgga 3’ |
|  | MM110R | 5’ atacacccgaaagggaaggaacgccg 3’ |
| Cy5-P_3778_ | MM110F | 5’ Cy5-aagttggtctcgaagcgcacgtcgc 3’ |
|  | MM110R | 5’ cagcaggcgctcgaggtcctcaa 3’ |
| **600-bp internal fragment of *nla28* in pMM301** | MM301F  MM301R | 5’ tggacagcatccagcagggcgcct 3’  5’ ccacccgcgtcttgaggtccttgtt 3’ |
| **Fragment for Nla28-DBD expression** | MM302F  MM302R | 5’ ctggcgctcaacgtgacgggcgt 3’  5’ acctgcaggctacgactcggcctccg 3’ |

**Supplementary Table S3.** Putative intragenic and intergenic σ^54^ promoters in *M. xanthus* PK/NRP gene clusters

| **Intragenic** | | | |
| --- | --- | --- | --- |
| **Regulated genes/operons** | **Functions** | **Putative promoter**  **−24/−12 sequence^a^** | **Position^b^** |
| MXAN1276 | Glutamate-cysteine ligase family 2 protein | GT**GG**CGCGGCCCTT**GA**A | -476 |
| MXAN1281 | hypothetical protein | CT**GG**AACGCGTGCA**GC**A | -712 |
| MXAN1286-1287 | ABC transporter/ATP-binding protein;  hypothetical protein | TT**GG**ACCGGCTCGT**GC**G | +15 |
|  |  | TT**GG**CGCGGAAGGC**GC**A | +111 |
| MXAN1287 | hypothetical protein | AT**TG**CCCGCGCCCT**GC**T | -308 |
|  |  | GA**GG**CCCAACTGTT**GC**G | -233 |
| MXAN1290-1291 | Phosphotransferase; non-ribosomal peptide synthetase | CT**GG**CACGTGACGT**GT**T | -285 |
|  |  | GT**GG**TGCGGCGGAT**GC**A | -488 |
| MXAN1292 | hypothetical protein | CT**GG**CGCGCGAGGA**AC**T | -521 |
| MXAN1293 | protoporphyrinogen oxidase | CC**GG**TACACACGTT**GG**T | -285 |
|  |  | GA**GA**CACCGGCGTT**GT**T | -324 |
| MXAN1560 | class I aminotransferase | GT**GG**CACGTCGCTG**GT**G | -284 |
| MXAN1567 | urea amidolyase-like protein | CT**GG**CGCGGTTGCT**GC**G | -407 |
| MXAN1567-1568 | hydrolase family protein;  LamB/YcsF family/allophanate | CT**GC**CACGCGCCTG**GC**G | -443 |
| MXAN1570 | class V aminotransferase | AT**GA**CTCGCAGCTT**GC**G | -293 |
| MXAN1573 | AMP-binding domain protein | CT**GG**CCTCCACGGT**GG**T | -409 |
| MXAN1574 | TfoX domain-containing protein | CT**GG**CGCGCGCCGG**GG**T | -212 |
| MXAN1584 | hypothetical protein | GT**GG**CGCGGCCGCT**GC**T | -182 |
| MXAN1587 | hypothetical protein | GT**GG**GACATCCGGT**GC**G | -146 |
| MXAN1588-1590 | Aminotransferase; putative para-aminobenzoate synthase, component I | GA**GG**CACGCCGCAC**GC**T | -122 |
| MXAN1591-1592 | hypothetical proteins | CT**GC**TCTGCACGGA**GC**T | -217 |
|  |  | CT**GG**AAGTGGACCT**GC**T | -229 |
| MXAN1595 | hypothetical protein | AT**GG**CACGGGGGAC**GA**A | -115 |
|  |  | GA**GG**ATGACCGGCT**GC**T | -413 |
| MXAN1599 | putative methyltransferase | GT**GG**CCAGCCGGGC**GC**G | -281 |
|  |  | GT**GG**TGCCGCTGCT**GC**T | -44 |
| MXAN1600 | class I aminotransferase | GA**GG**CCCGCTCCGA**GC**T | -402 |
|  |  | CT**GG**CTCGGGCGTT**GC**G | -270 |
| MXAN1601 | fatty acid desaturase family protein | GT**GG**CCCGGGCACT**GC**G | -384 |
| MXAN1603 | putative non-ribosomal peptide synthetase | GT**GG**CCTGGGTGGT**GC**C | +84 |
| MXAN1605 | putative permease | CT**GG**AAGGGTCGCT**GC**T | -265 |
| MXAN1606 | hypothetical protein | GT**GG**CCGCCTTCAT**GC**T | -260 |
| MXAN1608 | hypothetical protein | TA**GG**AACGCACGAC**CC**T | -286 |
| MXAN3460 | Gfo/Idh/MocA family oxidoreductase | CT**GG**TCCACATCAT**GC**T | -182 |
| MXAN3627 | hypothetical protein | AT**GG**CGGAAGAGCT**GC**T | -305 |
| MXAN3627-3628 | hypothetical protein; non-ribosomal peptide biosynthesis thioesterase | CT**GC**CACGCCAGCA**GC**T | -383 |
| MXAN3630 | polyketide synthase type I | CT**GG**CGGACATCTG**GC**A | -315 |
| MXAN3630-3631 | polyketide synthase type I | AA**GG**AACAGCTGTT**GC**G | -290 |
| MXAN3635-3636 | non-ribosomal peptide synthase/polyketide synthase | AA**GG**CGTGCGCATT**GC**T | -193 |
| MXAN3643-3644 | non-ribosomal peptide synthase; isochorismatase | GT**GG**AAAACCATCT**GC**T | -297 |
| MXAN3645-3647 | 2,3-dihydroxybenzoate-AMP;  isochorismate synthase;  2,3-dihydroxybenzoate-2,3- dehydrogenase | GT**GG**CACGCGAAAT**CC**C | -254 |
| MXAN3778 | DnaK family protein | TT**GA**GACGGAGATT**GC**G | -284 |
| MXAN3931 | hypothetical protein | GT**GG**GCCGGTTCCG**GC**T | -289 |
| MXAN3932 | polyketide synthase | CA**GA**CACGCCCATC**GC**T | -58 |
|  |  | TT**GA**AAAACCGGTT**GC**T | -75 |
| MXAN3933 | mixed type I polyketide synthase-peptide synthetase | GT**GG**TACTTCGCTT**AC**T | -222 |
| MXAN3935 | non-ribosomal peptide synthase/polyketide synthase Ta1 | CT**GG**CACGAACCCT**GG**G | -205 |
|  |  | TT**GG**CGCTGAGCGT**GC**C | -301 |
|  |  | TT**GG**CTCGCGTCTG**GC**T | -413 |
| MXAN3936 | polyketide synthase | TT**GG**TCCGCCGGGC**GG**T | -318 |
| MXAN3938 | polyketide synthase | CT**GT**CGCTCGTGAC**GC**T | -140 |
| MXAN3939-3941 | enoyl-CoA hydratase; polyketide beta-ketoacyl:acyl carrier protein synthase | CT**GG**CTCTGCAACT**GC**G | -229 |
| MXAN3943 | cytochrome P450 family protein | GT**GG**CGCATGCCTT**TC**T | -436 |
| MXAN3945 | polyketide TA biosynthesis protein TaF | CT**GG**ACAGCCTGCG**GC**T | -82 |
| MXAN3945-3946 | polyketide TA biosynthesis protein TaF; putative acyl carier protein | CT**GT**CGCGCGTGGT**GC**T | -205 |
| MXAN4000-4001 | non-ribosomal peptide synthase/polyketide synthase | CT**CG**CCCGCAGGTT**GC**A | -161 |
| MXAN4002 | nonribosomal peptide synthetase | AG**GG**CACGGCCCGT**GA**T | -451 |
| MXAN4290-4291 | putative thioesterase; hypothetical protein | CT**GG**AGCGCGTGCT**GC**T | -354 |
| MXAN4293-4295 | hypothetical protein; transporting ATPase;  patatin-like phospholipase family protein | AT**GT**CGCCGTGCTT**GC**A | -118 |
| MXAN4296 | non-ribosomal peptide synthetase | CT**GG**GAGTGGACCT**GC**T | -134 |
| MXAN4297-4298 | polyketide synthase type I | AT**GC**GTCGGCAGTT**GC**T | -364 |
| MXAN4299 | non-ribosomal peptide synthase/polyketide synthase | AT**GG**CGCTCGAGTT**GC**G | -164 |
| MXAN4300 | polyketide synthase type I | CT**GG**CGAAGCGGCT**GC**T | -167 |
| MXAN4413 | hypothetical protein | CT**GG**ACGACCGGTT**GG**T | -186 |
| MXAN4416 | cephalosporin hydroxylase family protein | CT**GG**GCCACGGATT**GC**T | -332 |
| MXAN4525-4526 | non-ribosomal peptide synthase; polyketide synthase type I | AT**GG**CGCTGGAGCT**GC**G | -243 |
|  |  | AT**GG**CGCTGCTGTT**GG**A | -359 |
|  |  | GT**GG**CAGGGCAGGT**GC**G | -452 |
| MXAN4527 | polyketide synthase | CT**GG**CTGAACAACT**GC**A | -156 |
| MXAN4598 | non-ribosomal peptide synthase | AT**GG**AACGGCGCAT**CC**T | -41 |
| MXAN4599 | M28 family peptidase | CT**GG**GCTACGGGGT**GC**T | -534 |
| MXAN4600 | radical SAM domain-containing protein | GT**GG**CCTGGGCGTC**GG**T | -493 |
| MXAN4602 | hypothetical protein | CT**GG**CCCGTCTGGA**GC**T | -241 |
| MXAN4604 | hypothetical protein | AT**GT**CGCTCGACTT**GC**T | -62 |
| **Intergenic** | | | |
| **Regulated genes/operons** | **Functions** | **Putative promoter^a^**  **−24/−12 sequence** | **Position^b^** |
| MXAN1284-1285 | 2-isopropylmalate synthase/homocitrate synthase family protein; tryptophan halogenase | AG**GG**CAAGGCATTT**CC**A | -121 |
| MXAN1563-1564 | alkyl hydroperoxide reductase C | CT**GG**CACGGTGACT**GC**T | -69 |
| MXAN1567-1568 | hydrolase family protein; LamB/YcsF family/allophanate | CT**GG**CACGCCAGCG**TC**T | -44 |
| MXAN1578 | metallo-beta-lactamase family protein | AC**GG**CGCAGCGCTT**GC**T | -45 |
| MXAN1579 | hypothetical protein | GG**GG**CGCAATCCTT**GC**G | -104 |
|  |  | GG**GG**CGCAGGTCTT**GC**G | -75 |
| MXAN3638 | M19 family peptidase | CT**GG**TACTTCGAGT**GC**A | -32 |
| MXAN4532 | non-ribosomal peptide synthase | GT**GG**CACAAGCTGC**GC**T | -173 |
| MXAN4600 | radical SAM domain-containing protein | GC**GG**TAAAGTCTTT**GC**T | -41 |

^a^-12 and -24 regions of putative σ^54^ promoters were identified in a previous study^37^.

^b^Distance between the −12 region of the putative σ^54^ promoter and the predicted initiation codon*.*

**Supplementary Figure S1**

**A.**


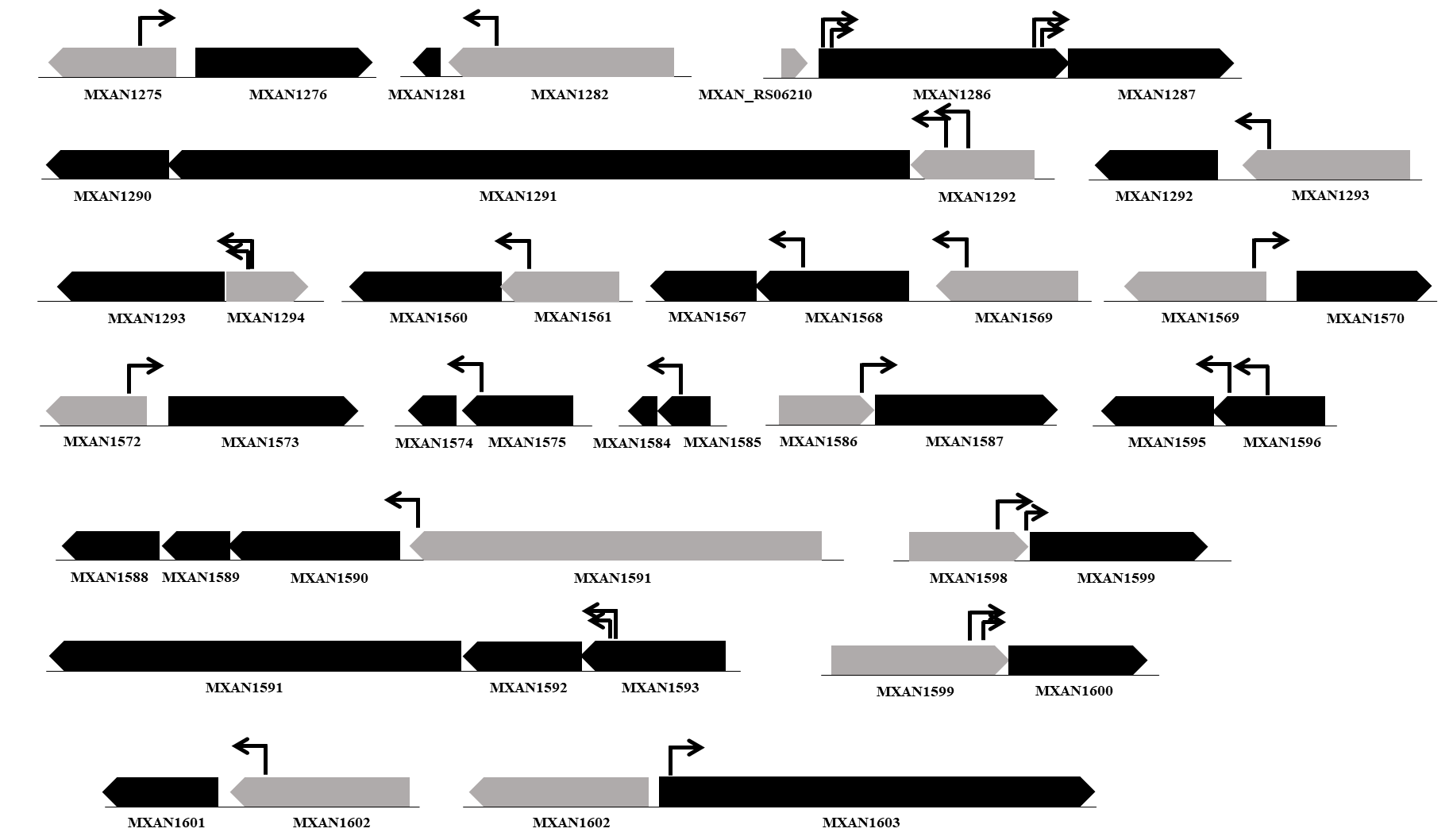


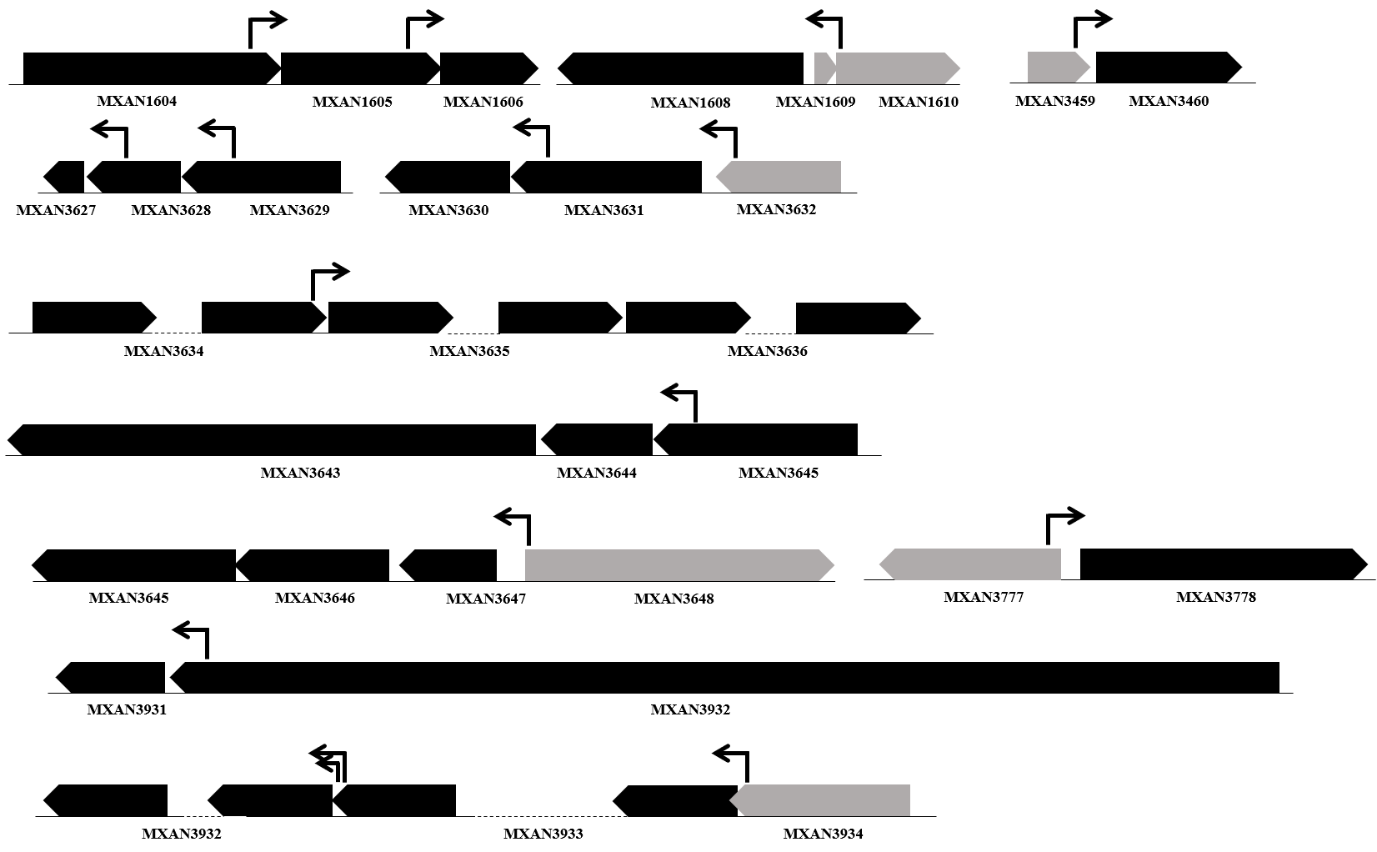


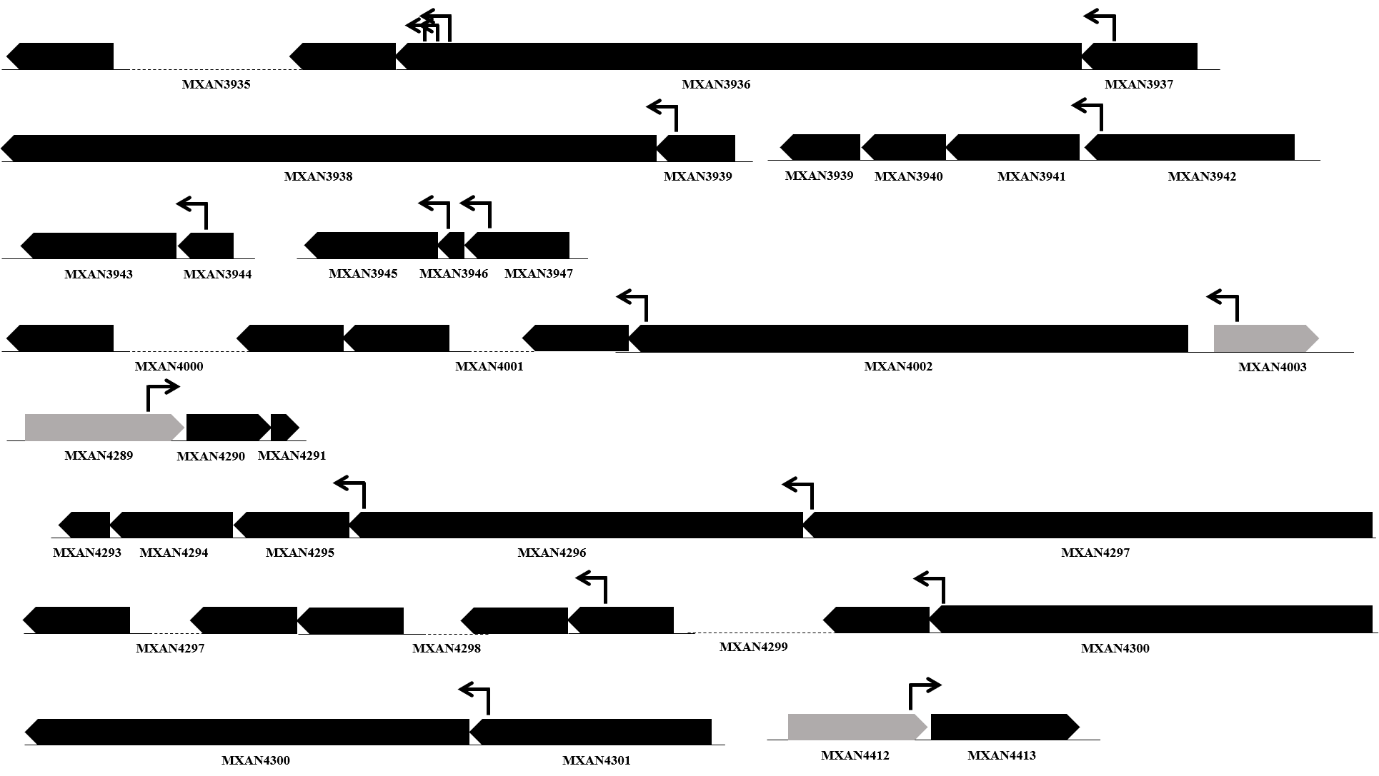


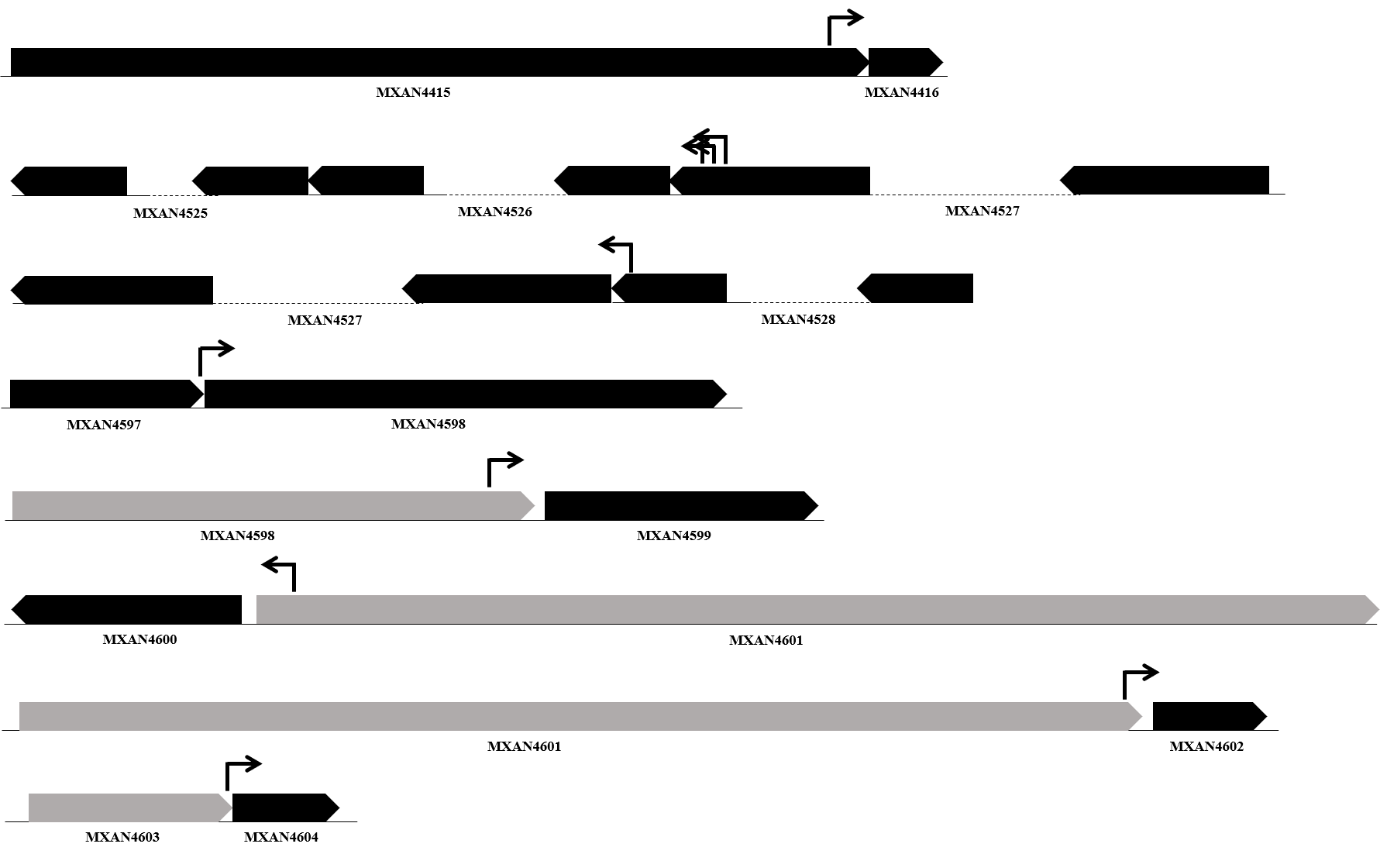


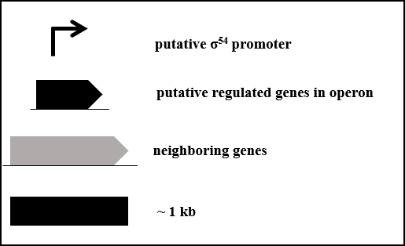


**B.**


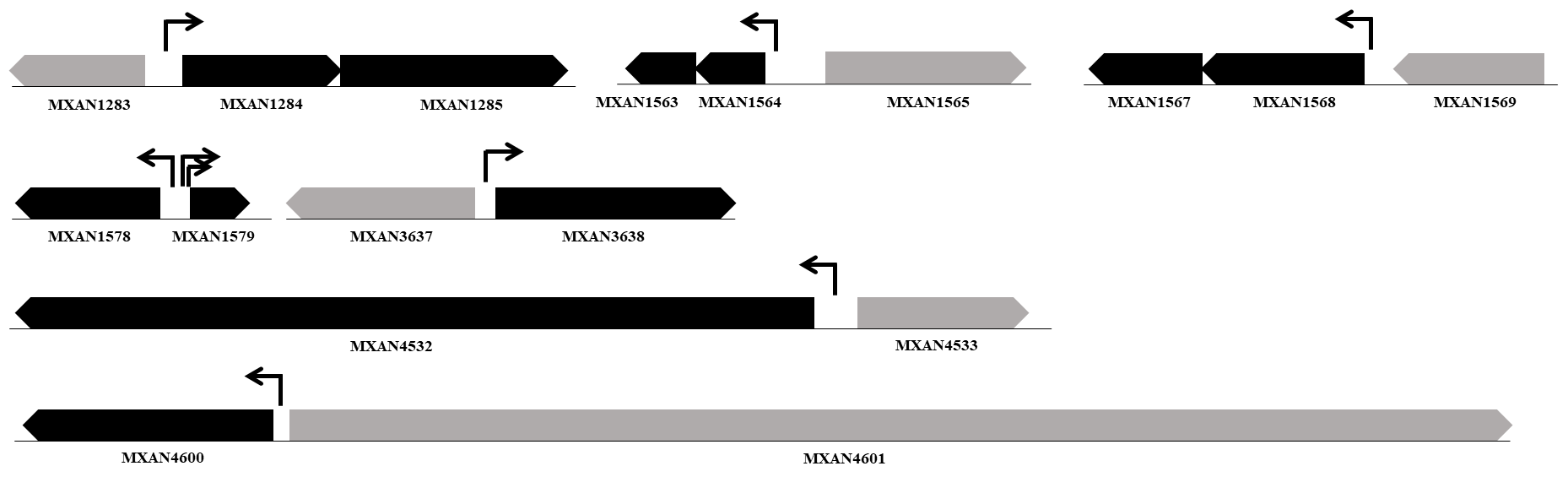


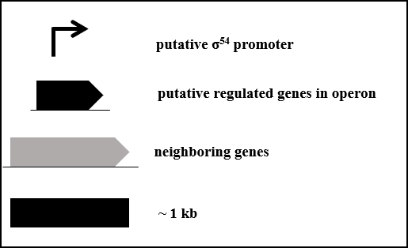


**FIGURE S1. Locations of putative σ^54^ promoters in *M*. *xanthus* PK and NRP gene clusters.** Of the 83 putative σ^54^ promoters identified in *M*. *xanthus* PK and NRP gene clusters^38^, 74 are predicted to be intragenic (A) and 9 are predicted to be intergenic (B).

**Supplementary Figure S2**


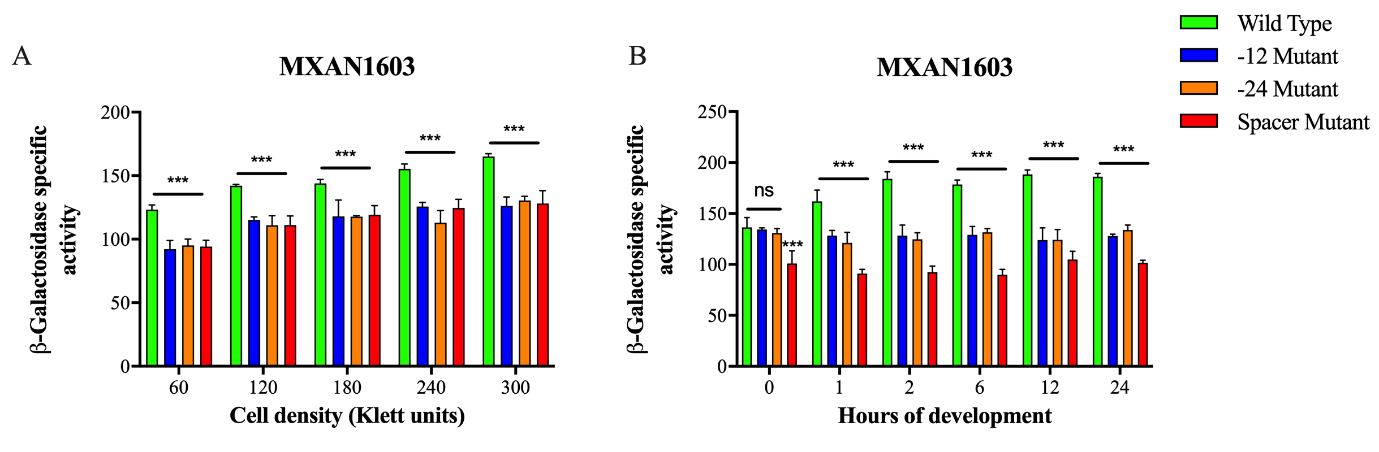


**FIGURE S2. In vivo activities of the wild-type MXAN1603 promoter and derivatives of the promoter carrying a mutation in the putative -12 region, -24 region or spacer region.** Wild-type and mutant fragments of the MXAN1603 promoter were cloned into a *lacZ* expression vector and transferred to the wild-type *M. xanthus* strain DK1622. At various cell densities during growth (A) and time points during development (B), β-galactosidase-specific activities in cells carrying a wild-type or a mutant promoter fragment were determined. (N=3 per group; Error bars: Mean ± SD; ***p < 0.001; **p < 0.01; *p < 0.05 for in vivo activities of mutant promoters versus wild-type promoters).
